# Texas State Medical Association

**Published:** 1900-03

**Authors:** 


### TEXAS STATE MEDICAL ASSOCIATION.

### Preliminary Announcement and Program of the Thirty-second Annual Meeting, April 24, 25, 26 and 27, at Waco.

COMMITTEES.

*Committee of Arrangements*—Dr. J. H. Sears and Dr. J. C. J. King, Chairmen; H. C. Black, C. T. Young, A. M. Curtis, G. B. Foscue, C. Guy Riely, J. R. Ferrell, J. T. Harrington, N. A. Olive, H. O. I. Halbert, A. J. Weathered, C. E. Smith, A. H. Snead, W. W. Wilkes, E. W. Sanderson, J. W. Hale, R. W. Park, J. A. Pipkin, W. F. Cole, J. J. Dean, T. C. Walker.

*Committee of Reception*—C. Guy Reily, Chairman; C. E. Smith, A. J. Cammack, J. T. Harrington, H. W. Brown, J. H. Sears, G. Jack, A. H. Snead, R. F. Minnock, E. W. Sanderson, A. J. Weathered, O. I. Halbert, G. B. Foscue, J. W. Hale, B. E. Hadra, W. O. Wilkes, T. C. Walker, W. W. Wilkes, J. T. Harrington, W. A. Howard, R. W. Park, N. A. Olive, A. M. Curtis, J. J. Dean, J. R. Ferrell, W. F. Cole, W. P. Griffith, O. L. West, G. C. McGregor, J. T. Parker, C. T. Young, J. A. Pipkin, H. C. Black, Jas. S. Collins, D. R. Wallace.

OFFICERS.

*President*—A. B. Gardner.......................... Bellville.
*First Vice-President*—B. E. Hadra...................... Waco.
*Second Vice-President*—Geo. H. Lee.................. Galveston.
*Third Vice-President*—F. D. Thompson............. Fort Worth.
*Secretary*—H. A. West............................ Galveston.
*Treasurer*—R. F. Miller............................ Sherman.
*Orator*—Joe Becton.............................. Greenville.

OFFICERS OF SECTIONS.

1. *General Medicine*—H. P. Cooke, Galveston, Chairman; S. C. Red, Houston, Secretary.
2. *Obstetrics and Diseases of Children*—J. J. Robert, Hillsboro, Chairman; Sam R. Burroughs, Buffalo, Secretary.
3. *Surgery*—B. F. Kingsley, San Antonio, Chairman; J. R. Stuart, Houston, Secretary.
4. *Medical Jurisprudence*—I. E. Clark, Schulenberg, Chairman; A. N. Denton, Austin, Secretary.

5. *State Medicine and Public Hygiene*—T. W. Shearer, Wallisville, Chairman; J. W. Scott, Houston, Secretary.

6. *Gynecology*—T. J. Bell, Tyler, Chairman; Clarence Warfield, Galveston, Secretary.

7. *Ophthalmology, Etc.*—R. H. Chilton, Dallas, Chairman; Jno. O. McReynolds, Dallas, Secretary.

8. *Microscopy and Pathology*—W. R. Blailock, Chairman; J. M. Frazier, Belton, Secretary.

### TRANSPORTATION.

The Association is to be congratulated upon the following liberal rates offered by the railroads for this meeting, affording an excellent opportunity to visit the beautiful and hospitable city of Waco:

*H. & T. C. and M., K. & T.*—From stations 75 miles from Waco, four cents per mile round trip; 100 miles and over 75 miles, $3.00 round trip; over 100 miles, one fare for round trip.

*S. A. & A. P., Cotton Belt, Texas Central.*—One fare for round trip.

Other roads connecting with the above will make the same proportionate rate.

### HOTEL RATES.

State House, $2.50 per day; National, $2.50 per day; Pacific, $2.00 per day; McLellan, $1.25 to $1.50 per day; Waverly, $1.25 per day; Exchange, $1.25 per day; St. Charles, $1.50 to $1.75 per day; Blackwell, $1.00.

### MEMORANDA.

1. Papers here announced will take precedence of those subsequently offered.

2. Papers will be read in the order as appears upon the program.

3. Papers read before the Association must be handed to the Secretary as soon as they shall have been read. Such papers, as well as those read by title, belong to the Association, but authors, if they so desire, may publish them elsewhere prior to publication in the Transactions.

4. Alterations will not be made in a paper after it is in type, except at the expense of the author.

5. Illustrations will be inserted only at the expense of author.

6. Papers read shall not occupy more than twenty minutes, except by permission. Discussion shall be limited to five minutes, unless the time be extended by unanimous consent of the sections, except as to the author of a paper, who shall have ten minutes in which to close.

No member shall speak more than once to the same subject without permission. (See By-Laws, Art. XV.)

PRELIMINARY PROGRAM.

The session will begin Tuesday morning at 11 o'clock. The place of meeting, the Auditorium. Members and applicants are requested to assemble early in order to register, as far as possible, before the opening exercises. The Secretary, Treasurer, and members of the Reception Committee will be present to aid registration. Applicants for membership who belong to district or county societies in affiliation with the State Association may join upon presentation of a certificate duly signed and accompanied with $5.00, one year's dues, and $2.50 initiation fee. Other applicants will sign blank form, giving post-office address and county. Each application must have the signature of two members and be accompanied by $7.50, covering one year's dues and the initiation fee. Such applicant must furnish diploma or other documentary evidence of graduation for inspection by the Judicial Council. Badges will be furnished members and applicants after registration.

In accordance with a resolution adopted at San Antonio, requiring that the time for the meeting of the several sections to be printed in the program, and that all papers in a certain section be presented in their regular order at the time specified, the following is submitted:

PROVISIONAL ROSTER.

FIRST DAY—*Tuesday, April 24, 11 A. M.*

Opening exercises as follows:

Call to order by Chairman Committee of Arrangements, Dr. J. H. Sears or J. C. J. King, Waco.

Invocation, Rev. Dr. Frank Page, Rector St. Paul's.

Welcome, by Mayor of Waco.

Welcome, by Hon. John G. Winter.

Welcome, by Dr. W. R. Blailock, McGregor, representing Central Texas Medical Association.

Response, Dr. A. B. Gardner, President.

Roll call.

Reading Minutes of Last Meeting.

Secretary's Annual Report, Dr. H. A. West.

Treasurer's Annual Report, Dr. R. F. Miller.

President's Annual Message and Recommendations, Dr. A. B. Gardner, Bellville.

*Afternoon Session, 2 P. M.*

Medical Section.

*Night Session, 8 P. M.*

Section on Obstetrics and Diseases of Children.

SECOND DAY—*Wednesday, 9 A. M.*

Executive Business.
Surgical Section.

*Afternoon Session, 2 P. M.*

Section on State Medicine and Public Hygiene.
Memorial Services.

*Night Session, 8 P. M.*

Section on Gynecology.

THIRD DAY—*Thursday, 9 A. M.*

Executive Business.
Section on Ophthalmology.

*Afternoon Session, 2 P. M.*

Appointment of Nominating Committee.
Election of Officers.
Medical Section Recalled.

*Night Session, 8 P. M.*

President's Address, Annual Oration, and Memorial Address upon Life and Services of R. M. Swearingen, by F. E. Daniel, Austin, at the Auditorium.

9:30 o'clock—Reception to Members and Accompanying Ladies.

FRIDAY, 27TH—*Morning Session.*

Papers not Previously Read in the Various Sections.
Executive Business.

GENERAL MEDICINE.

*Part I.*

1. Chairman's Address—"Treatment of Typhoid Fever," H. P. Cooke, Galveston.

2. "Observations Upon a Recent Epidemic of Scarlet Fever in Houston," S. C. Red, Houston.

3. "Diphtheria Treatment and Complications," Chas. C. Gidney, Granger.

4. "Croupous Pneumonia," T. M. Wilson, Thornton.

5. "Electricity as a Therapeutic and Diagnostic Agent," M. M. Smith, Austin.

6. "Leprosy—Report of a Case," Malone Duggan, Eagle Pass.

7. "Etiology of Dysentery in Adults," E. A. Walters, Crowley. To open discussion: T. N. Self, Joshua, and W. T. McNeal, Valley Mills.

8. "Stomach Diseases as now Understood," T. J. Bennett, Austin.

*Part II.*

Special Assignment.

9. "The Treatment of Tuberculosis with Watery Extract of Tubercle Bacilli," J. D. Osborn, Cleburne. To open discussion: J. C. Erwin, McKinney.

10. "Tuberculosis—Its Prevention and Treatment," D. R. Fly, Amarillo.

11. "Climatic Conditions in Western Texas, and Their Influence Upon Pulmonary Conditions," J. A. Rawlins, El Paso.

OBSTETRICS AND DISEASES OF CHILDREN.

1. Report of Chairman, J. J. Robert, Hillsboro.

2. "Anæsthesia in Labor," J. A. Rawlings, El Paso.

3. "Prevention of Fever Following Labor," T. J. Crofford, Memphis, Tenn.

4. "Ophthalmia Neuralisem," J. O. McReynolds, Dallas.

5. "Abortion—Report of a Case," J. E. Gilcreest, Gainesville.

6. "Deal with Vomiting in Pregnancy, Mechanically and Otherwise," Wm. T. Baldwin, Paris.

7. "Puerperal Infection—Treatment," E. E. Guinn, Rusk.

8. "Puerperal Fever—Treatment," J. C. Carlton, Bonham.

9. "Report of a Case of Cerebral Meningitis Following Measles—Recovery," H. A. West, Galveston.

SURGERY.

*Part I.*

1. Chairman's Report—"A Brief Review of the Progress of Surgery for the Past Year," B. F. Kingsley, San Antonio.

2. "Mortality in Appendicitis," Joseph Price, Philadelphia, Pa.

3. "Stone in the Bladder and Lithotomy," Frank Paschal, San Antonio.

4. "The Technique of Early Operation in the Treatment of Cleft Palate," J. E. Thompson, Galveston.

5. "Report of a Case of Renal Hyatids," J. B. Stinson, Sherman.

6. "Left Inguinal Colotomy—Report of a Case," E. B. Jackson, San Antonio.

7. "Report of Three Surgical Operations: (1) Excision of Upper Jaw; (2) Wiring of Patella; (3) Removal of Appendix—Recovery in Each," H. A. Barr, Beaumont.

8. "Three Cases of Closure of Jaws, Produced by Mercurial Stomatitis," J. E. Thompson, Galveston.

9. "Fractures of the Pelvis and Traumatisms of the Pelvis Viscera," R. Harvey Reed, Rock Springs, Wyo.

10. "Hemorrhoids," M. L. Langford, Baileyville. Discussion opened gy G. R. Tabor, Bryan.

11. "The Anatomy of the Renal Vessels in Relation to Digital Explorations of the Pelvis of the Kidney," W. Keiller, Galveston.

*Part II.*

Special Assignment.

12. "Treatment of Fracture in the Neighborhood of the Shoulder, Elbow, and Wrist Joints," Bacon Saunders, Fort Worth. To open discussion, I. P. Gunby, Sherman.

### STATE MEDICINE AND PUBLIC HYGIENE.

*Part. I.*

1. Report of Chairman, T. W. Shearer, Wallisville.

2. "Maladministration of Public Medical Affairs in the State of Texas," H. A. West, Galveston.

3. "Education, the Doctor's Duty and Opportunity," Jno. S. Lankford, San Antonio.

*Part II.*

Special Assignment.

4. "The Public Health and the State's Duty to Protect it," M. M. Smith, Austin. To open discussion: F. E. Daniel, Austin.

### GYNECOLOGY.

*Part. I.*

1. Report of Chairman—"How Much Have Obstetricians Contributed to the Work of the Gynecologist?" T. J. Bell, Tyler.

2. Suppurative Forms of Pelvic Inflammation," Joseph Price, Philadelphia, Pa.

3. "Extra Uterine Pregnancy," Henry Schwarz, St. Louis, Mo.

4. "Report of a Case of Ectopic Gestation," J. E. Gilcreest, Gainesville.

5. "Varieties of Pelvic Inflammation," T. J. Crofford, Memphis, Tenn.

6. "A Plea for the More Thorough Study of Hysteria in the Female," A. L. Norsworthy, Houston.

*Part II.*

Special Assignment.

7. "The Reflex Neuroses in Women," S. E. Hudson, Austin. To open the discussion: R. R. Walker, Paris.

OPHTHALMOLOGY, ETC.

*Part. I.*

1. Report of Chairman, R. H. Chilton, Dallas.

2. "Report of Cases Illustrating the Extremes in Special Surgery —Boldness and Conservatism," E. P. Daviss, Houston.

3. "Foreign Body in the Globe, with Extraordinary History," W. R. Thompson, Fort Worth.

4. "Preliminary Iridectomy for Senile Cataract," H. L. Hilgartner, Austin.

5. "The Use of Mercural as a Valuable Non-irritating Antiseptic in Intraocular Suppurative Processes," Joseph Mullen, Houston.

6. "Ulcerative Keratitis," R. F. Le Mond, Denver, Colo.

7. "The Present Status of Ear Work," R. F. Miller, Sherman.

8. "The Differential Diagnosis of Simple Conjunctivitis and Iritis," W. M. Moore, Paris.

9. "Sarcoma of the Naso Pharynx—Report of Cases," J. O. McReynolds, Dallas.

*Part II.*

Special Assignment.

10. "Eye Lesions of Syphilis," R. E. Moss,SanAntonio. To open discussion: H. L. Hilgartner, Austin.

The indications are favorable for a large attendance at Waco. The co-operation and attendance of all regular and reputable physicians in the State is earnestly solicited. The program is suggestive of a pleasant as well as profitable time.

Fraternally,

H. A. WEST, Secretary.

This edition only. Preserve your program.

